# Health Impact Assessments in Spain: Have They Been Effective?

**DOI:** 10.3390/ijerph17082959

**Published:** 2020-04-24

**Authors:** Maite Morteruel, Amaia Bacigalupe, Elena Aldasoro, Isabel Larrañaga, Elena Serrano

**Affiliations:** 1 Department of Nursing, University of the Basque Country (UPV/EHU), 48940 Leioa, Spain; 2 OPIK-Research Group for Social Determinants of Health and Demographic Change, University of the Basque Country (UPV/EHU), 48940 Leioa, Spain; amaia.bacigalupe@ehu.eus; 3 Department of Sociology 2, University of the Basque Country, 48940 Leioa, Spain; 4 Department of Health, Basque Government, 01010 Vitoria-Gasteiz, Spain; ealdasoro-san@euskadi.eus; 5 Department of Health, Basque Government, 20013 Donostia-San Sebastian, Spain; mlarranagapadilla@gmail.com (I.L.); esk-irun9@euskadi.eus (E.S.)

**Keywords:** health impact assessment, effectiveness, intersectoral action for health

## Abstract

Background: Health impact assessment (HIA) has scarcely been developed in Spain, in comparison with other European countries. Moreover, little is known about the effectiveness of HIA, taking into account direct impacts—changes on the decision-making process—as well as indirect impacts or those related to the process outcomes. From this broad perspective of HIA usefulness, the purpose was to assess the effectiveness of five HIAs carried out in Spain at the local level, and the role played by context and process factors on these impacts. Methods: We carried out a qualitative study based on 14 interviews to HIAs participants from different sectors. A documentary review and nonparticipant observation techniques were implemented for an in depth understanding. Results: The direct effectiveness of the HIAs was partial, but they had indirect effectiveness in all cases. The institutional and socio-political context, however, was not favorable to effectiveness. The elements of the process were largely determined by the context, although their influence, mediated by the role of proactive individuals, favored the effectiveness of the HIAs. Conclusions: When assessing HIA effectiveness, it is important to take into account a broad perspective on the nature of impacts and those factors influencing direct and indirect effectiveness. In Spain, the institutional and sociopolitical context was less favorable to HIA effectiveness than process-related factors. In order to implement the Health in All Policies strategy, will be necessary to improve context-related factors, such as institutional facilitators for HIA and democratic quality.

## 1. Background

Health and disease are mainly influenced by events that occur outside health systems and depend largely the social determinants of health (SDOH). These determinants refer mainly to people’s living and working conditions, which modulate their opportunities to achieve better health and wellbeing outcomes [1]. These, in turn, are determined by macroeconomic and social policies and by existing patterns of inequality or power hierarchies stratifying people by sex, age, place of origin, ethnic group, sexual orientation, and sexual identity [1]. There continues to be social inequality in health, resulting from the unequal and unfair distribution of SDOH and reducing such inequality is considered a political priority [2]. Efforts to tackle events that threaten the sustainability of life, such as the spread of non-communicable diseases, climate change, rapid urbanisation and economic crises are associated with a high level of uncertainty and need agreements between different agents, from global to local levels, and from governments to communities [3].

Good governance of health involves acting to respond to these challenges, advocating for the development of coordinated and integrated action at all levels and across all areas of the government, as well as in other sectors. Health in All Policies (HiAP) is based on this approach and means systematically taking health implications into account in public policy planning [3]. Intersectoral action for health is a way to advance the HiAP strategy, and according to Shankardass et al. [4], it is characterised by different levels or typologies of work ranging from the exchange of information, cooperation and coordination between sectors, to the integration of structures and budgets.

The local level of government has great potential to influence SDOH [5] and, therefore, a good context for putting HiAP into practice, given its functions in areas such as employment, urban planning, housing and transport. In recent years, as shown by the Healthy Cities program, local policies have gone beyond sectoral logic towards more cross-sectoral plans that are integrative and participatory [6]. This can be seen in urban renewal planning in Europe, which show progress towards a more comprehensive approach to the shaping of urban spaces, with the consideration of socioeconomic factors [7] as well as the participation of local stakeholders [8].

Health impact assessment (HIA) is an approach that may catalyze the implementation of intersectoral action for health at the local level [9]. It brings into dialogue decision-makers, key informants and the community to assess the impact on health of policies (plans or projects) that are not directly health related and envision possible solutions that maximize their positive and minimize their negative impact on health [10]. In this sense, as a tool aligned with the values of good governance for health—equity, participation, democracy, evidence-based policy making, transparency and sustainability [11]—has been shown to be successful in challenging the political agenda [12].

The Merseyside guidelines [13] describe the development of HIA as involving five procedures, culminating in evaluation of processes and monitoring of adherence to recommendations. Nevertheless, as well as usually being at an early stage of development, evaluation of HIAs has primarily been focused on the process, specifically, its ability to predict impacts [14]. Although HIAs have demonstrated usefulness in these areas, the emerging need to justify the allocation of resources to HIAs has led to an increase in the number of HIA evaluations, as well as a broadening of the concept of effectiveness associated with HIAs [14]. This conceptualization underlines, on the one hand, that HIAs have a wide range of impacts beyond those originally described, and on the other, that there are multiple factors that determine HIA impacts and that have yet to be defined [15]. Elliot and Francis [16] introduced the concept of HIA “indirect impacts”, referring to generated knowledge, tools, skills and relationships between agents from different areas or fields and between authorities and community. Hence, we can refer to a narrow [17] and a wider perspective on the effectiveness of HIAs.

Narrow approaches to HIA effectiveness include the conceptual frameworks of Parry and Kemm [18] and Wismar et al. [19], the latter being more commonly used. Wismar et al. defined effectiveness as the ability of HIAs to influence the decision-making process and be taken into account by decision-makers to modify decision-making [19]. Regarding the wider perspective, the most notable example is the framework of Harris-Roxas and Harris [14] that includes the direct effectiveness of HIAs, namely, their impact on decision making, as well as their indirect effectiveness, in particular, their influence on the decision-making process through the resulting learning and their contribution to advancing intersectoral action. Additionally, this wider perspective suggests that we explore factors related to the HIA process, as well as those that outline the institutional and socio-political context of decision-making. In this sense, relevant analyses have aimed at the HIA challenges to fit public policy development [20]. Haigh et al. [21] reviewed and confirmed this framework for describing HIA effectiveness, but also built on it, adding some cross-sectional factors to those identified in the original, including proactive positioning and the role of timing.

In Spain, some HIA-related legislative advances have occurred in the last years. The current national General Public Health Law [22], as well as many regional laws, encourage this type of assessment, and it has become imperative in some cases in Andalusian region. Beyond this legislative progress it remains a poorly used tool in the Spanish context, with the exception of a few studies carried out voluntarily and within research projects. Some HIA experiences have been developed based on the SDOH model and with an equity focus, and some others relaying the environmental risk approach. Between 2006 and 2014, five HIAs were completed based on the SDOH model and with an equity focus and following the Merseyside guidelines. They were related to urban planning -four urban regeneration projects: Bilbao HIA [23], Barceloneta HIA [24], Alcalá de Guadaíra HIA [25] and Bay of Pasaia HIA [26]; and one city master plan: Vitoria-Gasteiz HIA [27]. The urban regeneration projects were undertaken in urban areas considered socioeconomically disadvantaged compared to the rest of the city.

From a health promotion perspective and the will to advance towards implementing intersectoral action for health in Spain, we considered that it would be useful to assess the effectiveness of these five HIAs in Spain, as well as the contextual and process factors that have facilitated or hindered them, to advance implementation of HIAs in particular, and that of a HiAP approach in general, both in Spain and internationally.

Due to the interest in the HiAP, the general aim of this study was to assess the effectiveness of the five HIAs carried out in Spain from an SDOH perspective, based on the methods of the Merseyside guidelines, as well as identify the most important determinants of their effectiveness. The specific objectives of this study were: (1) to describe and characterize the direct and indirect 146 effectiveness of HIAs according to the conceptual framework of Haigh et al.; and (2) to assess the determinants of HIA effectiveness according to the conceptual framework of Haigh et al., including both context and process factors, such as cross-sectional factors (corresponding to the individual sphere), that had an impact on this effectiveness.

## 2. Methods

The study was designed to analyze the five HIAs effectiveness based on qualitative methods. The main characteristics of the HIAs analyzed are summarized in Table 1, as well as the role of the interviewees in each. The study variables, related to effectiveness and its determinants, were selected based on the framework proposed by Haigh et al. [21]. Table 2 indicates the criteria used to assess the presence of various determinants of effectiveness.

**Figure 1:**
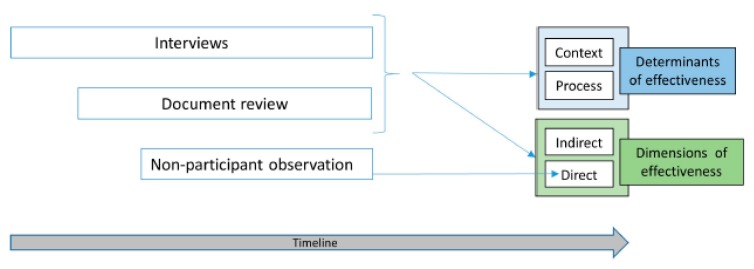
Diagram of the whole analysis process.

Three types of techniques for data collection and analysis were used, the second and third types enabling to investigate in more depth and complete information collected using the first type—qualitative analysis, shown in Figure 1. The way methods contributed to different results is collected in Table 3.

(1) Individual interviews with civil servants or politicians who participated in any of the HIAs or who had influence over or responsibility for decision-making in the projects are shown in Table 3. Purposive sampling was conducted, based on the intention of engaging informants from the health sector (or individuals responsible for the HIA) and from other sectors (participants in the HIA or nonparticipating decision-makers) for each HIA. Next, informants were added by snowball sampling, considering adequacy of the sample size to achieve data saturation, seeking diversity in points of view, and ensuring appropriateness of the individual (in terms of sector and role). The respondents were approached by telephone and mail. Finally, 14 interviews were carried out between October and December 2016, following a semi-structured interview guide (Supplementary Materials) based on Haigh et al.’s framework addressing both direct and indirect dimensions of HIA effectiveness and the context and process-related determinants of each HIA effectiveness. For direct effectiveness, the informants were asked how many of the recommendations of the HIA were finally carried out. For indirect effectiveness and the determinants of effectiveness, Haigh et al.’s categories related to learning and other distal impacts, as well as context and process aspects influencing HIAs effectiveness were tested. The interviews lasted approximately one hour and were conducted by the first author, who had training in interviews and focus groups. The major part of the interviews was conducted face-to-face at the respondents’ workplace. Written informed consents were obtained from all the interviewees and a processing of personal data were registered. The interviews were transcribed and manually analyzed through a thematic analysis. Haigh et al.’s categories were tested against the themes that were emerging. For each HIA, an explanatory framework about its effectiveness and its determinants was created and finally produced a list of the dimensions, categories and elements identified across the cases to compare them with each other.
(2) Review of a range of different types of documents shown in Table 3 published—from 2005 to 2017 in English, Catalan or Spanish—and nonpublished. Search terms were related to the interventions denominations and the search databases were institutional websites, documentation centers, scientific meetings reports, social media and online press. The criterion for including the documents was that they contained descriptions, analysis or interpretation of the HIAs under analysis or related to the projects associated with the HIAs. Relevant information about the dimensions not fully covered by the interviews was collected. In addition, the document review provided a situated knowledge about each HIA context, that contributed to improve the analysis of interviews.
(3) Nonparticipant observation in two settings (Bilbao and Barceloneta) where the HIAs were carried out, seeking to access information related to the impact of the recommendations of the HIA that we failed to obtain from the in-depth interviews. Observations, based on structured checking lists, were held in December 2016 by both the first and second authors in the case of Bilbao and by the first author in the case of Barceloneta. In the case of Bilbao, the places and features along the neighborhood that were built or modified by the regeneration project, such as the new elevators, the speed reduction elements, the new park, the accessibility improvements in the civic center, were observed. In the case of Barceloneta, features in the intervened blocks—such as elevators and renewed fronts—were checked.

The information obtained was synthesized in tables. On the one hand, information concerning the direct and indirect effectiveness of the HIAs is given in Table 4. The information on direct effectiveness was summarized as a percentage, considering only the projects that were finally carried out, dividing the number of recommendations implemented (considering those for which the implementation has been reported) by the total number of recommendations made. The indirect effectiveness was analyzed for each of the categories identified, qualitatively assessing the level of the impact achieved by each HIA. 

On the other hand, we assessed the association between each type of effectiveness and the information obtained related to determinants. First, we identified the complete (“Yes”) or “partial” presence or absence (“No”) of determinants of the effectiveness in each case, using a categorization explained in Table 2. Second, we described the contribution of each determinant to each type of effectiveness (direct and indirect) of each HIA, as shown in Table 5. Specifically, green was used to indicate factors that contributed positively to the effectiveness of the HIA; yellow to indicate those that neither favored nor hampered its effectiveness, and finally, red to represent those that had a negative impact on its effectiveness.

## 3. Results

This study provides two types of results: on the one hand, regarding the effectiveness of the HIAs and, on the other, factors that determine this effectiveness, summarized in Table 4 and Table 5, respectively.

Regarding direct effectiveness shown in Table 4, all the HIAs were followed by implementation of at least some recommendations, although the rate of adherence varied. The scope of the interventions adopted following the recommendations and hence the resulting changes in SDOH also varied between the HIAs. The changes made to the urban environment related to the Bilbao and Bay of Pasaia HIAs were substantial, as were the measures to improve employability and social inclusion in relation to the Alcalá de Guadaíra HIA.

All the HIAs had indirect effectiveness, as presented in Table 4. The greatest positive impacts were seen in the “learning” category: technical learning, related to skills to undertake HIAs, as reported by all HIA teams; social learning, observed in association with the establishment of intersectoral actions, highlighting key factors for bringing sectors together; and also conceptual learning, related to a social model of health, as revealed in the interviews with the individuals from sectors other than health.

“As well as finally producing a series of recommendations, there was another objective, namely, learning and skills development and the handling of the tool […] certainly, this second objective was achieved to a greater extent than that related to the recommendations themselves”.

“We learnt that public health should abandon its traditional role of health authority in the context of intersectoral work”

Health sector HIA promoter, Bay of Pasaia HIA

“Urban projects used to be developed from purely urban perspective. If we seek to improve people’s quality of life, we should take a more comprehensive approach, considering equity, equality, and gender criteria, and all the better if we think about this before the project”

Environmental sector technician, Vitoria-Gasteiz HIA

Moreover, the relationship between health and other sectors was seen to improve—compared to the previous situation—, especially in the case of urban planning. Furthermore, all interviewees referred to other actions following from the HIAs, related to: the sharing of what had been learnt and the establishment of new lines of work to promote HiAP in regional government; the creation of a nationwide HIA association; the incorporation of HIA into legislation for the first time in a Spanish region; the involvement of the health sector in intersectoral actions across municipal authorities; and the commissioning of new HIAs.

“The HIA provided a new focus, which had not been considered until now, and was useful for establishing collaborations with certain sectors, such as urban planning”

Health sector HIA promoter, Vitoria-Gasteiz HIA

“Now there is a greater involvement of the public health authority in the community, as an entity that can collaborate in certain fields, and that has something to say”

Health sector HIA promoter, Bay of Pasaia HIA

“If this HIA was seen to be effective in some way, it was at the regional government level, when it came to encouraging, [and] teaching by showing the intervention to loads of people. In this sense, it did have an impact; I think, in fact, it had a big impact”

Health sector technician, Bilbao HIA

Concerning the contextual determinants of effectiveness shown in Table 5 and, specifically, the institutional context, the uptake of the SDOH model served as an “umbrella” framework for launching the HIAs. In turn, the HIAs themselves contributed to awareness of the discourse concerning SDOH, facilitating the emergence of other actions from within government. Nevertheless, in general, this institutionalization of the social model of health was not accompanied by a political mandate supporting the values of the HIA. This limited political backing, together with the vertical structure and working patterns of the public administration, and the lack of a culture of intersectoral collaboration and public participation in government affairs, restricted both the ambition of the HIA and its execution in accordance with these values.

“We worked under the framework of the health plan; one of its objectives was to reduce inequalities in health through interventions that evaluated the impact of non-health interventions on health equality. This was ideal”

Health sector technician, Bilbao HIA

“It seemed to us that there was insufficient institutional support and political commitment [...]. Commitment is about assuming that failure is part of the process and a prerequisite for future success. So, I think that there is a long way to go at this level, even though it is included in the health plan...”

Health sector HIA promoter, Bay of Pasaia HIA

“In the public administration, the organizational structure is very hierarchical, with very distinct levels, and we are very used to working vertically, and when we have to work horizontally, we find it difficult. We need to speak the same language and have a shared vision and common goals from the outset. This is about working by project rather than by management body”

Participation sector technician, Barceloneta HIA

Various elements of the socio-political and economic context related to the economic crisis, changes in political leadership and existing tensions between citizens and government, as well as between institutions responsible for decision-making, interfered in the effectiveness of the HIAs. On the one hand, they slowed processes down, and on the other, they made it more complicated to set up meetings and agreements with key agents, as well as build public participation. In particular, existing tensions made it difficult for public participation to result in the emergence of health-related issues, these competing against other issues related to the tensions and demands not met by the government.

“The economic crisis [...], changes in political leadership at the local and regional levels [...] made the process difficult. They disrupted things and delayed us a lot, because they extended the time frame; we had to raise the question of the HIA with the various urban planning promoters and decision-makers all over again”.

“Every meeting [achieved] with the institutions was a victory, because they felt judged in some way [...]. The course of these projects was so complex and contentious, that it hampered transparency between the agents involved [...]. Moreover, it caused a wave of mistrust and skepticism among community about everything proposed by the government that made it difficult to be brought over discussion, reflection, participation”

Health sector HIA promoter, Bay of Pasaia HIA

With regard to decision-making context, positive institutional relationships between the parties involved in the HIAs favored a good level of understanding and the recognition of common goals and shared interests among key agents. This total or partial alignment of goals, although opportunistic in nature in some cases, facilitated working together on the HIAs, as well as their direct effectiveness. On the other hand, the lack of formal agreements on the HIAs and lack of alignment of projects with the HIA values hindered their effectiveness. Specifically, the lack of political support for the concept of participation, above all when there were socio-institutional tensions, hampered efforts to encourage participation and effective inclusion of its results in the HIAs.

“[The lack of a formal agreement] made the follow-up difficult given the changes in political leadership and meant that the follow through on the recommendations was based on the good personal relationships established”

Health sector HIA promoter, Alcalá de Guadaíra HIA

“We disguised it as a research project to soften its image compared to what an HIA is expected to do, namely, empower, enable real participation, etc. so that they would let us do it [...] it was more technical than political in nature”

Health sector technician conducting Bilbao HIA

Concerning key elements of the HIAs that contributed to their effectiveness, we should highlight first, the use of scientific evidence and a diverse range of knowledge. The use of evidence from the scientific literature increased the legitimacy and acceptability of recommendations. Nevertheless, the contextualization of this information with local evidence, in particular, the description of the historical and socio-political context and exploration of the perceptions of people involved, was considered necessary, when there was a little or no scientific literature from settings near or similar to those of the HIAs.

Furthermore, the participation of experts with different profiles in the HIAs (architects, engineers, specialists in qualitative methods, etc.) improved the quality of the HIAs and contributed to the identification of the impacts and the drafting of recommendations.

“It’s not clear to me that here people who shop in supermarkets have a poorer health than those who shop in local shops. I am not convinced that the evidence generated in the United Kingdom is generalizable to our setting”

Health sector technician conducting Bilbao HIA

“It is essential to analyze and understand your context; if you don’t know it well enough, it’s going to be difficult to explain and manage future problems or difficulties, and it’s unlikely that the HIA will be effective”

Health sector HIA promoter, Bay of Pasaia HIA

As a second key element, the allocation of funding to the HIAs enabled them to be performed according to the goals set. In some cases, resource constraints determined the intensity of intersectoral work, as well as the extent of public participation. Nonetheless, a greater quantity of resources was not associated with greater effectiveness of the HIA if there was a lack of other elements such as political support. In relation to this, HIAs being flexible and adaptable to both the resources available and contingencies that occur in the decision-making process was perceived as a third key element for effectiveness. In view of limited resources, the use of simple tools to bring a health perspective to decisions and the use of pre-existing structures and procedures were cited as potential good practices that could be used as an alternative to a full HIA.

“In an HIA, we need to take into account time frames, in urban planning [...] interests converge, there is a need for time for all the procedures, legislative requirements [...] and it’s subject to twists and turns. Changes that considerably complicate matters and that can seriously slow the process. This translates into long waits and uncertainty for the community. HIA must bear in mind this impact on citizens and, at the same time, should adapt its own development to those circumstances”

Health Sector HIA promoter, Bay of Pasaia HIA

“We must manage to develop efficient and practical tools that provide substantive knowledge and added value to the process that already exists, that are easy to use, such as evidence summaries [...] enabling integration of the HIA into procedures that are already carried out or health issues into other consultation procedures”

Health sector technician conducting Alcalá de Guadaíra HIA

With regard to the HIA procedures, a more in-depth development of the assessment was associated with greater effectiveness. In particular, the involvement and participation of key decision-making agents made it more likely that recommendations would be taken into account. HIAs which had active and stable involvement of such individuals achieved greater direct and indirect effectiveness.

“I think that if we don’t establish a mutual understanding with decision-makers and obtain their commitment, the HIA might be completed, but the process would be much more tortuous and the implementation of the recommendations more difficult”

Health sector HIA promoter, Bay of Pasaia HIA

“It is important that decision-makers are present, since this ensures that what has been drafted can be carried over to the design of the project”

Environmental sector technician, Vitoria-Gasteiz HIA

“We executed the plan, made the investment, they commissioned a different plan and there we left it. Perhaps we failed to create strong enough links with the units at the municipal level responsible for implementing this type of policy for there to be follow through on these recommendations, beyond this specific project”

Participation sector technician, Barceloneta HIA

For its part, the process of achieving public participation contributed to indirect effectiveness in several ways. Firstly, the population concerned received information about projects, as well as the roles, competencies and interests of institutional and social agents in the projects. This helped improve transparency and bring polarized parties together, especially communities and local councils. Secondly, the participating population were given an overview of the social model of health and issues related to social inequalities in health in their area. Thirdly, the public participation generated recommendations to respond to the needs and opportunities for action identified.

“It was an interesting insight into how people see health impacts. They made proposals beyond the two specific interventions, which have also recently been accepted and budgeted for; in this sense, participation has been essential”

Health sector HIA promoter, Bay of Pasaia HIA

“Thanks to the HIA, people have learnt about the characteristics of the projects, the positions of different parties [...]. No one changed their opinion, but they did improve their understanding other points of view”

Health sector technician conducting Bay of Pasaia HIA

Lastly, transparency during the process and accountability, as evidenced in the follow-up and monitoring of the HIA recommendations implemented, were elements that made HIAs more effective. Firstly, the lack of an agreed plan for the implementation of the recommendations was sometimes cited as a barrier to effectiveness. Secondly, monitoring the implementation of the recommendations on the ground by HIA teams had a positive impact on members of the community, as well as on the institutional plan.

“[Monitoring on the ground] is what little by little may consolidate these intersectoral relationships and rebuild relationships where there have been tensions, because if not, we came, we asked questions and that’s it. Here we are and the health sector is presenting issues playing a role in the citizens’ everyday life. After two years, we want to know if there has been any change since that experience”

Health sector HIA promoter, Bay of Pasaia HIA

Furthermore, individual proactiveness was a factor that cut across the analyzed elements. It contributed to the effectiveness of the HIAs by, among other things: creating momentum for HIA initiatives; helping the finding of common ground and an understanding between those involved in working together; executing the HIA as far as possible in accordance with the values of the process, especially in cases of a lack political support, with few resources or a history of tensions; identifying funding to make continuity of the HIA viable; and sharing and disseminating information about the HIA experience and the knowledge acquired.

## 4. Discussion

This is the first study assessing the effectiveness of HIAs conducted in Spain from an SDOH perspective. The assessment has been performed based on a broad conceptual model of HIA effectiveness. The local nature of the HIAs studied, all being related to urban planning and regeneration, has enabled comparisons. Further, the study has employed key methodological tools of analysis developed in other settings and has suggested others, such as the use of the framework of the determinants of health inequality [1] to assess the impact of HIAs on these factors, as well as the typology of intersectoral work of Solar et al. [4] to assess the progress made in this area.

The study has revealed some degree of direct effectiveness in cases in which the integrated urban regeneration projects were finally undertaken. The degree of change in the SDOHs since implementation of the recommendations was variable. In the case of indirect effectiveness, we found benefits from most of the dimensions of the HIAs. The categories of “learning” and “impact on other actions” had the greatest positive impact.

Regarding determinants of HIA effectiveness, factors related to the institutional context or health governance were, in general, unfavorable for the effectiveness of the HIAs analyzed. The social-political and economic context of the projects and the HIAs had a negative impact on effectiveness, since it made the HIAs more complex and threatened their viability. The decision-making context was favorable in cases in which there was alignment of values, goals and objectives. With regard to the process, the use of evidence and a diverse range of knowledge, availability of appropriate resources for the necessary development of the HIA, and flexibility in this development contributed to the effectiveness. Greater involvement and participation of key decision-making agents and community, together with transparency and accountability played an important role in direct and indirect effectiveness. Finally, proactive behavior was a catalyst for HIA effectiveness in multiple ways.

In general, these findings are in agreement with the main reviews of evaluations of HIA effectiveness, bearing in mind that most adopted a “narrow” view of effectiveness. Regarding direct effectiveness, Dannenberg [35] found that about half or more of the HIAs reviewed ended up having an impact on decision-making. Rhodus et al. [36] and Bourcier et al. [37] noted the effective role of HIAs in the integration of health-related issues into decision-making processes.

In relation to indirect effectiveness, the factors most commonly identified have been conceptual learning—more related to the model of SDOH [17,36] than to social inequalities in health [37]—and the strengthening of intersectoral action [35]. In terms of determinants of effectiveness, our results highlighted two facilitators of HIAs: the presence of political commitment, related to the institutional context; and shared spaces and language, related to structures, as well as a public health culture at the national level, these being consistent with the findings of Davenport et al. [38] and Wismar et al. [19].

As in our study, they found that these factors had a significant impact on values, goals and objectives established in the HIA [17]. In relation to the decision-making context, the positive influence of values and objectives common to all the stakeholders on effectiveness was an element highlighted by Haigh et al. [17]. A good relationship and understanding between the participating sectors, as well as the agreements established for the HIA were identified as elements that facilitated effectiveness.

As for the process, the lack of scientific literature in the Spanish context about interventions carried out on social determinants and health and their impacts on health was also considered as a challenge for the future in other evaluations of HIAs [35]. Similarly, as reported previously [36,37,38] the availability of resources was identified as a key element for effectiveness and, to an even greater extent, the adaption of the HIA to the existing resources. With respect to procedures, public participation has been described as an element that facilitates HIA effectiveness [21], given that it provides and allows the use of “high-quality evidence” [36]. In the present study, participation generated essential evidence for the identification of opportunities and health needs and was, on its own, a source of direct and indirect effectiveness, given the impact on the community of having engaged and receiving feedback on the information obtained [39].

Finally, we should highlight the individual dimension, an element that original framework authors encouraged, to be considered in future HIA effectiveness analyses [14]. In our case, as Haigh et al. purposed [21], individual proactiveness was a cross-cutting element that enabled HIAs viability and to achieve the very best of them in terms of direct and indirect effectiveness.

In this study, we have identified elements notably several associated with the Spanish economic, socio-political and governance context, namely, the economic crisis, which had a specific origin and consequences that particularly affected the urban planning sector; the political situation, in which an HiAP strategy has not been adopted by political consensus [40]; and social and institutional tensions, resulting from very established political behaviors characterised by a democratic deficit and political disaffection. This is well illustrated by one of the cases analyzed, in which the HIA investigated the impact of taking no action in an area that had been abandoned and left deteriorating due to a lack of political agreement. Our findings highlight the value of the HIAs as a tool to press for action in the face of unmet needs that may be important for health.

### Limitations

One of the limitations of the study relates to the lack of scientific consensus on the conceptualization of the effectiveness of HIAs and its determinants. This study used the model corresponding to a wider view of effectiveness—collecting information on direct and indirect impacts—and their determinants related to the HIA process and the decision-making context. This set of factors allowed us to address questions inherent to the Spanish context that had not previously been analyzed in relation to HIA effectiveness.

On the other hand, the framework had limitations in capturing the degree to which HIAs were effective for systematically including equity in health in decision-making. By adopting a set of criteria, we attempted to assess the presence of this approach in the HIAs and its role in their effectiveness. Secondly, there were methodological limitations related to access to information. In some cases, it was not possible to obtain data that were needed to fully assess the direct and indirect effectiveness. Some measures taken in the projects were likely to be governed by laws and regulations in the corresponding field, and hence, it was not possible to determine whether or not their application was attributable to the HIA. In other cases, we were unable to obtain information regarding the implementation of recommendations or progress associated with the HIA, especially those with a social nature and those related to indirect effectiveness when it was not possible to contact the relevant people in the city council or other government authority. Additionally, a lack of data precluded assessment of the final execution of some interventions, and in turn, assessment the impact of the HIAs. Similarly, we were not able to assess the impact of any of the HIAs on direct health outcomes, because monitoring and evaluation of this type of impact was not carried out in the HIAs. This is another limitation associated with the nature of HIAs as research projects; a limited time frame did not allow the completion of the last phase of the HIA—monitoring and evaluation. To solve the problem of the lack of information regarding the adoption of certain recommendations concerning urban planning, we resorted to nonparticipant observation, by which we succeeded in filling some of the gaps. Another methodological limitation was related to the fact that interviews were significantly delayed in the cases of four of five HIAs, and this may have influenced the perception of the people involved. Lastly, the interview transcriptions and the analysis conclusions were not returned to the respondents to validate them.

Future research should be focused on investigating the perception of people living in the communities involved in the HIA. This would give a valuable perspective on aspects of the effectiveness of the HIAs not explored in this study.

## 5. Conclusions

When assessing HIA effectiveness is important to take into account a broad perspective about the nature of impacts and also those factors influencing direct and indirect effectiveness. In Spain, institutional and sociopolitical context was less favorable to HIA effectiveness than process-related factors. This result indicates the emergence of a new democratic culture for health, with implications for the public sector, focused on advancing the integration of the social model of health and effective sharing of political responsibility for health. This implies the promotion of governmental actions that are progressively less sectorial and more integrated, as well as the provision of sustainable structures for intersectoral work. Similarly, the use, validation and adaption of context related tools, such as HIAs, should be promoted at the political level, encouraging commitment and agreements between stakeholders. Transparency and accountability must be ensured in the execution and evaluation of these assessments, as well as being integrated as values into public service. Writing the use of HIAs into legislation might give momentum to their use and maximize their potential, but to these ends, we should not forget the role of awareness raising and efforts to advance in the implementation of intersectoral action for health. Finally, we should work on setting up innovative ways to enable effective public participation and strengthen the participatory culture in government and society.

## Figures and Tables

**Table 1:** Basic characteristics of the health impact assessments (HIAs) analyzed.

	UBC (Bilbao) HIA	Vitoria-Gasteiz HIA	Barceloneta HIA	Alcalá de Guadaíra HIA	Bay of Pasaia HIA
**Project framework**	Comprehensive reform plan for the neighborhood Uretamendi-Betolaza bypass (Bilbao)	Construction works associated with an urban masterplan involving burying the railway tracks underground as they pass through the city	Comprehensive intervention plan (neighborhood law) for the Barceloneta neighborhood (Barcelona)	Regeneration measures (with in the “Plan Urban” project) for the San Miguel neighborhood in Alcalá de Guadaíra	Masterplan for redevelopment of the Bay of Pasaia
**Year**	2005–2006	2007	2008–2009	2010	2012–2013
**Interventions analyzed**	-Improvements in access within the neighborhood and to the rest of the city accessibility -Redevelopment of various areas in the neighborhood and creation of green spaces	-Construction of new structures and development of services -Planning of uses for the space gained	-A comprehensive housing rehabilitation program	-Improvements in access to the neighborhood -Redevelopment of a street	-Construction of a new wholesale fish market, freeing up ground-level space -No action being taken to regenerate an abandoned area

**Table 2:** Criteria for evaluating the presence of determinants of effectiveness of each HIA.

Determinants of Effectiveness (Haigh et al., 2015)	Present?		
	Yes	Yes, Partially	No
***Decision-making context***			
**Alignment of the project and HIA values**	Alignment of all values	Alignment of some values	No alignment of values
**Alignment of the project and HIA formal objectives**	Alignment of all objectives	Alignment of some objectives	No alignment of objectives
**A suitable stage of planning of the project in order to make recommendations**	The project is well defined: it is easy to make recommendations	The project is poorly defined: it is difficult to make recommendations	The project is not defined: no recommendations can be made
***Key elements for the HIA***			
**Based on available evidence and knowledge**	Availability of several types of evidence, knowledge and skills necessary for the HIA	Lack of certain types of evidence, knowledge or skills necessary for the HIA	Complete lack of evidence, knowledge or skills for the HIA
**Economic resources**	Sufficient economic resources to fully the execute the HIA	Limited economic resources, constraining the execution of the HIA	No economic resources to execute the HIA
***Procedures***			
**Participation of key agents in decision-making**	Participation of key agents in the steering committee throughout the process	Limited participation of key agents in the process	No participation of key agents
**Community participation**	Community representation in the HIA steering committee or extensive community participation	No community representation in the HIA steering committee and limited community participation	No community participation
**Transparency and accountability in the HIA**	Full process transparency between authorities and towards the community and a plan to follow-up on recommendations	Limited process transparency and/or follow-up of recommendations without an established plan	No transparency or follow-up

**Table 3:** Methods and main contributions to results.

HIA	Interviews Role in HIA		Main Contributions to Results	Document Review	Main Contributions to Results	Nonparticipant Observation	Main Contributions to Results
	Role in HIA	Professional Position When HIA (Current Position, If Different)					
**Bilbao**	1.Health sector technician conducting HIA	1. Public Health technician at the autonomous government (University lecturer)	-Evidence to verify the direct and indirect HIA effectiveness and its determinants	-Health Plan of the Basque Country [28] -HIA-related scientific articles and institutional reports [23,29] -Historical documents -News articles -Social platforms websites	-Evidence to verify the contextual and process-related determinants of HIA effectiveness	-Work carried out on the regeneration plan in accordance with the HIA recommendations	-Evidence to verify the direct HIA effectiveness
**Vitoria-Gasteiz**	1.Health sector HIA promoter 2.Environmental sector HIA technician	1. Head of Public Health Department at the local level 2. Environmental Health technician at the local level	-Evidence to verify the indirect HIA effectiveness and its determinants	-Health Plan of Vitoria-Gasteiz [30] -HIA-related documents and institutional reports [27]	-Evidence to verify the contextual and process-related determinants of HIA effectiveness	-	-
**Barceloneta**	1.Health sector HIA promoter 2.City Development sector HIA participant 3. Citizen Participation sector HIA participant	1. Public Health technician at the local level 2. City Development technician (Municipal District Manager) 3. Citizen Participation technician (Director of a Municipal District Department)	-Evidence to verify the direct and indirect HIA effectiveness and its determinants	-HIA-related scientific documents and institutional reports [24] -Neighborhoods Plan documents -Autonomous Neighborhoods Law [31] -Historical documents -News articles -Social platforms websites	-Evidence to verify the contextual and process-related determinants of HIA effectiveness	-Work carried out on the regeneration plan in accordance with the HIA recommendations	-Evidence to verify the direct HIA effectiveness
**Alcalá de Guadaíra**	1. Health sector HIA promoter 2. Health sector technician conducting HIA	1. Director of Public Health institution at the autonomous government (Public Health consultant) 2. Public health technician at the autonomous government (University lecturer)	-Evidence to verify the direct and indirect HIA effectiveness and its determinants	-HIA-related scientific articles and institutional reports [25] -Regeneration Plan documents -Autonomous HIA legislation [32] -News articles	-Evidence to verify the contextual and process-related determinants of HIA effectiveness	-	-
**Bahía de Pasaia**	1. Health sector HIA promoter 2. Health sector technician conducting HIA	1. Deputy Director of Public Health at the autonomous government 2. Public Health technician at the autonomous government	-Evidence to verify the direct and indirect HIA effectiveness and its determinants	-Health Plan of the Basque Country [28] -HIA-related scientific articles and institutional reports [26,33,34] -Historical documents -News articles -Social platforms websites	-Evidence to verify the contextual and process-related determinants of HIA effectiveness	-	-

**Table 4:** Direct and indirect effectiveness of the health impact assessments (HIAs).

	Bilbao HIA	Vitoria-Gasteiz HIA	Barceloneta HIA	Alcalá de Guadaíra HIA	Bay of Pasaia HIA
DIRECT EFFECTIVENESS					
Total number of recommendations adopted	5/23 (21.7%)	.	5/8 (62.5%)	11/19 (57.9%)	3/26 (11.5%)
Additional to the projects	1/7 (14.3%)	.	2/3 (66.6%)	2/4 (50%)	2/11 (18.2%)
Regarding the design of the projects	4/7 (57.1%)	.	3/5 (60%)	2/3 (66.6%)	1/14 (7.1%)
Regarding the construction work phase	?	.	.	7/12 (58.3%)	0/1 (0%)
Changes in social determinants of health					
Physical environment/housing	+++	.	+	.	++
Employability, social inclusion and cohesion	?	.	.	++	?
INDIRECT EFFECTIVENESS					
Learning					
Conceptual	✓	✓✓	✓	✓✓	✓
Technical	✓	✓	✓	✓	✓
Social	.	✓✓	✓	✓	✓✓
Strengthening of intersectoral action	.	✓	✓	✓	✓✓
Impact on other actions	✓	.	✓	✓✓	✓
Other indirect impacts	✓	.	.	✓	✓✓

(.): no recommendations/impacts; (?): impact was unknown; (+): some changes; (++) marked changes; (+++) very marked changes (✓) some impact; (✓✓) marked impact.

**Table 5:** Determinants of the effectiveness of the HIAs.

Determinants of the Effectiveness of the HIAs		Bilbao			Vitoria-Gasteiz			Barceloneta			Alcalá de Guadaíra			Bahía de Pasaia		
		Present?	DE	IE	Present?	DE	IE	Present?	DE	IE	Present?	DE	IE	Present?	DE	IE
**CONTEXT**	**Institutional context for the HIA**															
	Institutionalization of the social model of health	**Yes**			**Yes**			**Yes**			**Yes**			**Yes**		
	Political commitment to the HIA	**No**			**Yes**			**No**			**No**			**No**		
	**Socio-political and economic context**															
	Economic crisis	**No**	**/**	**/**	**Yes**			**No**	**/**	**/**	**Yes**			**Yes**		
	Electoral situation	**No**	**/**	**/**	**No**	**/**	**/**	**No**	**/**	**/**	**Yes**			**Yes**		
	Social and institutional conflicts	**Yes**			**No**	**/**	**/**	**Yes**			**Yes**			**Yes**		
	**Decision-making context**															
	Understanding between the stakeholders	**No**			**Yes**			**Yes**			**Yes**			**No**		
	Formal agreement on the HIA	**Yes**			**Yes**			**No**			**No**			**No**		
	Alignment with HIA values	**No**			**Yes**			**Partial**			**Partial**			**No**		
	Alignment with HIA objectives	**No**			**Yes**			**Partial**			**Partial**			**Partial**		
	Good timing for the HIA	**Yes**			**Partial**			**Yes**			**Yes**			**Yes**		
**PROCESS**	**Key elements of the HIA**															
	Evidence and knowledge available	**Yes**			**Partial**			**Partial**			**Yes**			**Yes**		
	Sufficient resources for the HIA	**Yes**			**Yes**			**Partial**			**Partial**			**Yes**		
	Flexibility and adaptability of the HIA	**Yes**			**Yes**			**Yes**			**Yes**			**Yes**		
	**Procedures**															
	Involvement and participation of key agents	**Partial**			**Yes**			**Yes**			**Yes**			**Partial**		
	Community participation	**Yes**			**No**			**Partial**			**Yes**			**Yes**		
	Transparency and accountability	**Partial**			**Partial**			**Partial**			**Partial**			**Partial**		
	**INDIVIDUAL DIMENSION (CROSS-CUTTING)**															
	Individual agency and proactiveness	**Yes**			**Yes**			**Yes**			**Yes**			**Yes**		

DE: Direct effectiveness; IE: Indirect effectiveness; (Green): it contributed positively to the effectiveness of the HIA; (Yellow): it did not have an impact on the effectiveness of the HIA (either positive or negative); (Red): it had a negative impact on the effectiveness of the HIA; (/): Non-applicable

## Supplementary Materials

The following are available online at https://www.mdpi.com/1660-4601/17/8/2959/s1. Interview guide. The supplementary material presents the key questions addressed in the interviews in order to evaluate the direct and indirect effectiveness as well as the determinants of effectiveness of the HIAs.
